# References for Haplotype Imputation in the Big Data Era

**DOI:** 10.4172/2168-9547.1000143

**Published:** 2015-10-31

**Authors:** Wenzhi Li, Wei Xu, Qiling Li, Li Ma, Qing Song

**Affiliations:** 1 Center of Big Data and Bioinformatics, First Affiliated Hospital of Medicine School, Xi’an Jiaotong University, Xi’an, Shaanxi, China; 2 Cardiovascular Research Institute and Department of Medicine, Morehouse School of Medicine, Atlanta, Georgia, USA; 3 4DGenome Inc, Atlanta, Georgia, USA

**Keywords:** Imputation, References, Haplotypes

## Abstract

Imputation is a powerful in silico approach to fill in those missing values in the big datasets. This process requires a reference panel, which is a collection of big data from which the missing information can be extracted and imputed. Haplotype imputation requires ethnicity-matched references; a mismatched reference panel will significantly reduce the quality of imputation. However, currently existing big datasets cover only a small number of ethnicities, there is a lack of ethnicity-matched references for many ethnic populations in the world, which has hampered the data imputation of haplotypes and its downstream applications. To solve this issue, several approaches have been proposed and explored, including the mixed reference panel, the internal reference panel and genotype-converted reference panel. This review article provides the information and comparison between these approaches. Increasing evidence showed that not just one or two genetic elements dictate the gene activity and functions; instead, cis-interactions of multiple elements dictate gene activity. Cis-interactions require the interacting elements to be on the same chromosome molecule, therefore, haplotype analysis is essential for the investigation of cis-interactions among multiple genetic variants at different loci, and appears to be especially important for studying the common diseases. It will be valuable in a wide spectrum of applications from academic research, to clinical diagnosis, prevention, treatment, and pharmaceutical industry.

## Imputation

**Figure 1:**
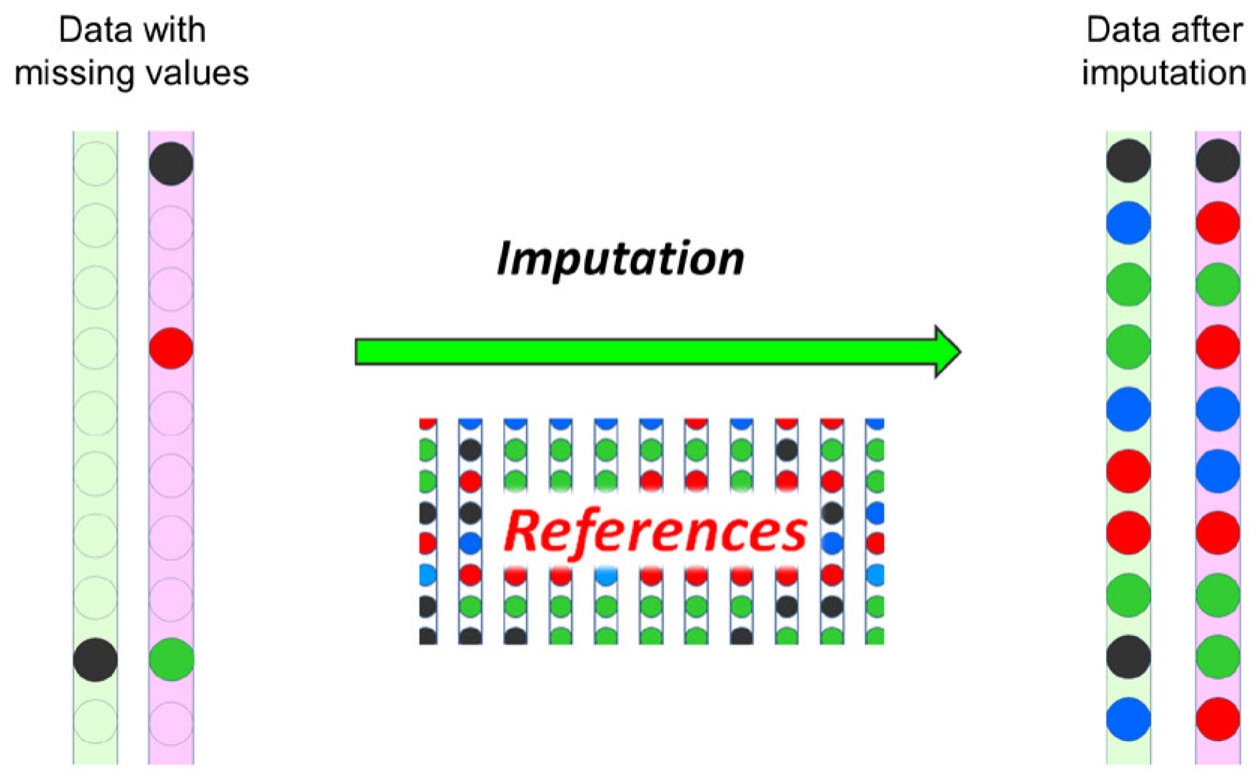
An illustration of the imputation Imputation is an in silico technology for replacing missing data with substituted values. The filled circles indicate the known data, the dotted unfilled circles indicate the missing value. After imputation, all missing values are inferred, and the data is complete. References are usually required for carrying out an imputation. Missing data can be a serious impediment for subsequent data analysis, thus it is critically important in the big data era.

In the big data era, as the number and size of genomic datasets enormously grow, people will regularly encounter a limitation on missing information. Since the missing data can adversely affect downstream analysis, how to deal with the missing values in the big datasets is emerging as a new and fast-moving research focus. Imputation is one of the most useful strategies for filling in those missing values using computational algorithms and large reference datasets (Figure 1) [1,2]. Imputation has been used widely in the analysis of genome-wide association studies (GWAS) to boost power, fine-map associations and facilitate the combination of results across studies using meta-analysis [3]. Without imputation, many gene associations would not be discovered in GWAS.

Solving the missing-value problem by imputation is a notoriously resource-demanding task. This process usually requires a reference panel, which is a collection of data from which the missing information can be extracted or imputed. Haplotype imputation requires ethnicity-matched references composed of known haplotypes (phased genotypes) in matched populations. A mismatched reference panel will significantly reduce the quality of imputation results [4,5] and yield false positive results [6]. However, currently available references only cover a limited number of ethnicities. The lack of ethnicity-matched references in many populations has severely restricted the use of big datasets in the research and development in these populations. It is important to establish the reference panels in a broad range of ethnic populations in the world for haplotype imputation.

It is well-known that different subpopulations have different unique SNPs (single-nucleotide polymorphisms) and different haplotypes due to their unique bottleneck events in ancient histories. For example, the intra-continental variation within the African populations is much greater than inter-continental populations. The population should not be described merely as “African”, “Sub-Saharan African”, “West African”, or “Sierra Leonian”, since each of those designators encompasses many populations with different geographic ancestries [7–9]. In the United States, the African-American population is featured by substantial admixture of multiple ancestral origins within various African populations and between different continental populations. Latino-Americans, American-Indians, and other minority populations are also featured by substantial admixture between various populations. This admixture feature reinforces the importance of establishing references of various ethnic populations.

Theoretically, several approaches can be used for obtaining the reference panels for haplotype imputation. First, people may carry out experimental haplotyping to establish the haplotype references to cover more ethnic populations. Second, people may pool together experimental haplotypes from various ethnic populations and use the pooled references for the imputation. Third, people may use the haplotypes with missing data from the imputation target population as the internal reference for imputation. Last, people may extract the information from the existing big data and create the reference panels for additional ethnic populations.

## Establishing reference panels by molecular haplotyping

Intuitively, a straightforward strategy to expand the haplotype references is to recruit human population samples from a wide-range of ethnic diversities and determine their molecular haplotypes. Molecular haplotypes can be determined either by high-throughput technologies [5,10–17] or by inference from trio genotypes [18]. Currently the reference datasets for haplotype imputation can be downloaded from the HapMap project and the 1,000 Genomes Project (KGP), in which the haplotypes are inferred either by the Mendelian Law of Inheritance or by statistical inferences. Since the launch of the International HapMap project in 2001 and the 1,000 Genomes project in 2008, totally 27 populations have been recruited, in which 17 populations have trios. Although trio haplotyping can reliable yield accurate chromosomal haplotypes except those triple-heterozygous sites, it is often unrealistic due to the difficulties to recruit pedigree specimens [18].

Alternatively, people may also recruit more samples and determine their molecular haplotypes experimentally using those cutting-edge technologies [10, 12–16, 19–21]. At present, sequencing technologies are still far from being able to construct long haplotypes directly through overlapping of those sequencing reads [12,17,22]. Single-sperm approach [23] needs sperms and can be used only on males. Single-chromosome isolation approach [10,13,14] has not been completely automated. Experimental haplotyping is still expensive and time-consuming for the data generation for establishing the reference panels in a diverse range of ethnic populations.

## Establishing reference panels by pooling haplotypes from multiple populations

It has been proposed to mix the haplotypes from some of available ethnicities to create a pooled reference panel (also called cosmopolitan reference panel) when an ethnicity-matched reference panel does not exist [4,5]. Indeed, it has been reported recently that pooled reference panels could give acceptable results [4]. However, this approach also suffers from some limitations.

One limitation is that this strategy requires *a priori* knowledge for identifying the major contributors and primary components before creating the corresponding pooling reference panel and for optimizing the mixing recipe on the number of haplotypes from each of available ethnicities [4]. The imputation accuracy of this strategy heavily depends on the optimization of the mixing recipe; a non-optimally mixed reference panel will reduce the imputation accuracy. For example, a study showed that the highest imputation accuracy may be as high as 97.8% (the Basque population imputed with a reference panel consisting of 48 CHB+JPT haplotypes, 120 CEU haplotypes, and no YRI haplotypes); and may be as low as 78.2% when the San population was imputed with a reference panel consisting of the entire CHB+JPT panel of 180 haplotypes [4]. In another study, it was noticed that it seems to be unpredictable what rationale to pool the ethnicities will be the best for imputation accuracy [24]. For example, when the sample was ASW, the [YRI+CEU] reference panel performed better than cosmopolitan reference [YRI+MKK+GIH+MEX+CEU]; interestingly, when the internal reference was involved, the largest cosmopolitan reference panel [ASW+CEU+YRI+MKK+GIH+MEX] performed the worst, but a reference panel pooled by the seemingly unrelated cohorts [JPT+CHB] performed the best [24]. It is unclear how this approach works for many untested populations and subpopulations yet. Theoretically only the cohorts from the ethnic populations that contribute to the admixture of the study population should be included in the pooled reference panel; however, a cosmopolitan panel does not always compromise the quality of imputation. Another potential limitation is the computing speed, the larger number of ethnicities in the pooled reference panel, the higher computer burden for using this cosmopolitan panel for imputation in reality. This is an important issue that should not be ignored in the big data era.

## Using internal reference panels for imputation

Another strategy is to use internal reference panels when an ethnicity-matched reference panel does not exist. It has been proposed to use the information of phylogenetic diversity from mathematical phylogenetic and comparative genomics to generate the most diverse internal reference panel efficiently, which has been reported to be able to substantially improve the imputation accuracy compared with randomly selected reference panels [25].

This strategy can avoid the substantial mismatch in ancestral background between the study population and the reference population. In addition, this strategy may combine the internal haplotypes with an available external panel to create a single cosmopolitan reference panel, so it can take the benefits from both of the existing big datasets contributed by the large genome projects as an external panel and the greater genetic similarity of the internal panel to the study population [26]. However, researchers may not always have sufficient study budget to create the internal reference panel with adequate sample size. When internal references are limited, the combination with external references should be careful; the choice of the existing external cohort for the augmentation of a small internal reference panel will be critical for the quality of imputation. A study showed that compared with the external-reference-only panel, augmenting an internal reference panel with a cosmopolitan external panel may considerably lower the imputation accuracy especially and interestingly when the ethnic backgrounds of augmented external references are related to the study population [24]. When a study does not have a budget for creating the internal references, an external reference panel will be the only choice.

## Establishing reference panels by statistically converting the genotypes into haplotypes

It has been well-known that unmatched reference panel will lower the quality of imputation. However, it is unrealistic to recruit a well-matched reference panel for every population in the world. Fortunately, enormous amounts of unphased genotype data have been generated and are still being generated by genome-wide SNP microarrays and whole-genome sequencing projects, these datasets have a broad representation of ethnic populations in the world. If these unphased genotype datasets can be converted and used as the reference panels for haplotype imputation, it will be a labor and cost-efficient strategy to quickly expand the ethnic representation for haplotype imputation.

In this approach, the unphased genotypes are first converted to haplotypes by a software tool based on statistically inferences [27–30]; and then the statistically resolved haplotypes are used as references for data A recent study showed that with the reference panel composed of statistically converted haplotypes from unphased genotypes, the imputation accuracy was 99.43 ± 0.05%, which is comparable with the imputation accuracy with the reference panel composed of molecular haplotypes (99.49 ± 0.05%) [31]. Even when as high as 50% values are missing in a dataset, this reference panel could still yield 98.5% imputation accuracy [31,32]. The quality of imputation was consistent across different study populations in this study [31]. This result demonstrates the feasibility of converting currently existing big data of unphased genotypes to be reference panels for high-quality imputations.

**Figure 2:**
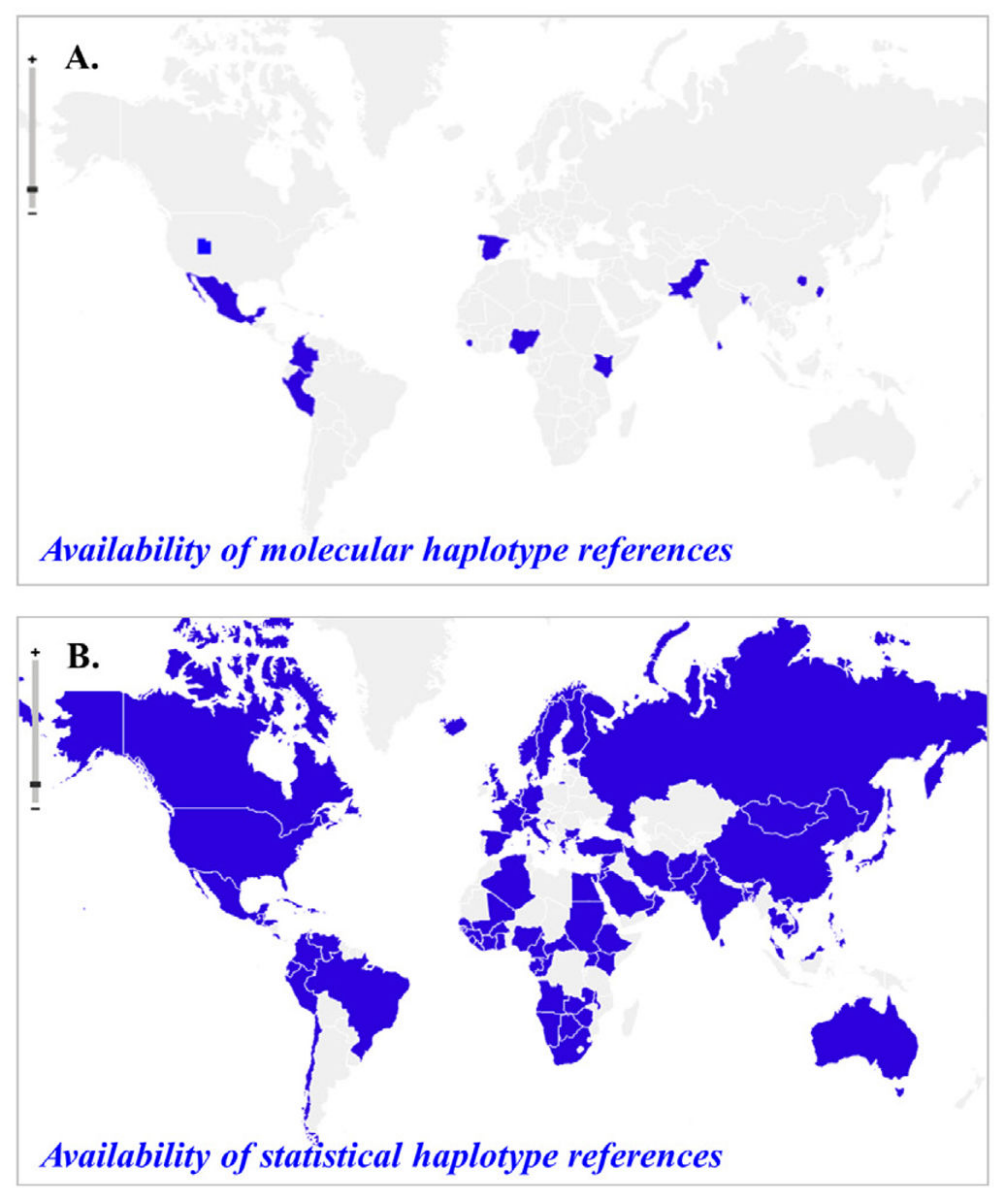
A geographic map of ethnic groups covered by statistically converted reference panels (A) The molecular haplotypes are composed of trio haplotypes from the HapMap project and the 1,000 Genomes Project (KGP). (B) The statistical references are composed of statistically resolved haplotype from unphased genotypes obtained from genotyping and next-generation sequencing platforms. The data is mainly retrieved form dbGaP, African Genome Variation Project, Human Genome Diversity Project (HGDP), AADM and GALA. The map was generated with “openheatmap” software.

This strategy has the potential to efficiently increase the coverage of ethnic diversities in the world (Figure 2) [31]. At the present time, the high-throughput experimental approach is still expensive for whole-genome haplotyping and has not generated any large dataset yet that can be used for imputation as references; all of those existing big datasets of molecular haplotypes were obtained by deducing the personal haplotypes from genotypes of trios [18,33]. So far, large genome projects, such as The International HapMap Project, The 1,000 Genomes Projects, African Genome Variation Project, and Human Genome Diversity Project (HGDP), have performed SNP genotyping on only a small number of human populations, totally 17 populations have trio genotypes. However, meanwhile, with the effects of whole-genome analysis such as genome-wide association studies (GWAS) and with decreasing cost of high-throughput microarray and sequencing technologies and other technologies, enormous amounts of unphased genotype data have been and are being generated. More than 1,000 big datasets have been collected and organized. Even the datasets in the dbGaP database cover a large diversity of ethnicities (Supplementary Table 1).

**Figure 3:**
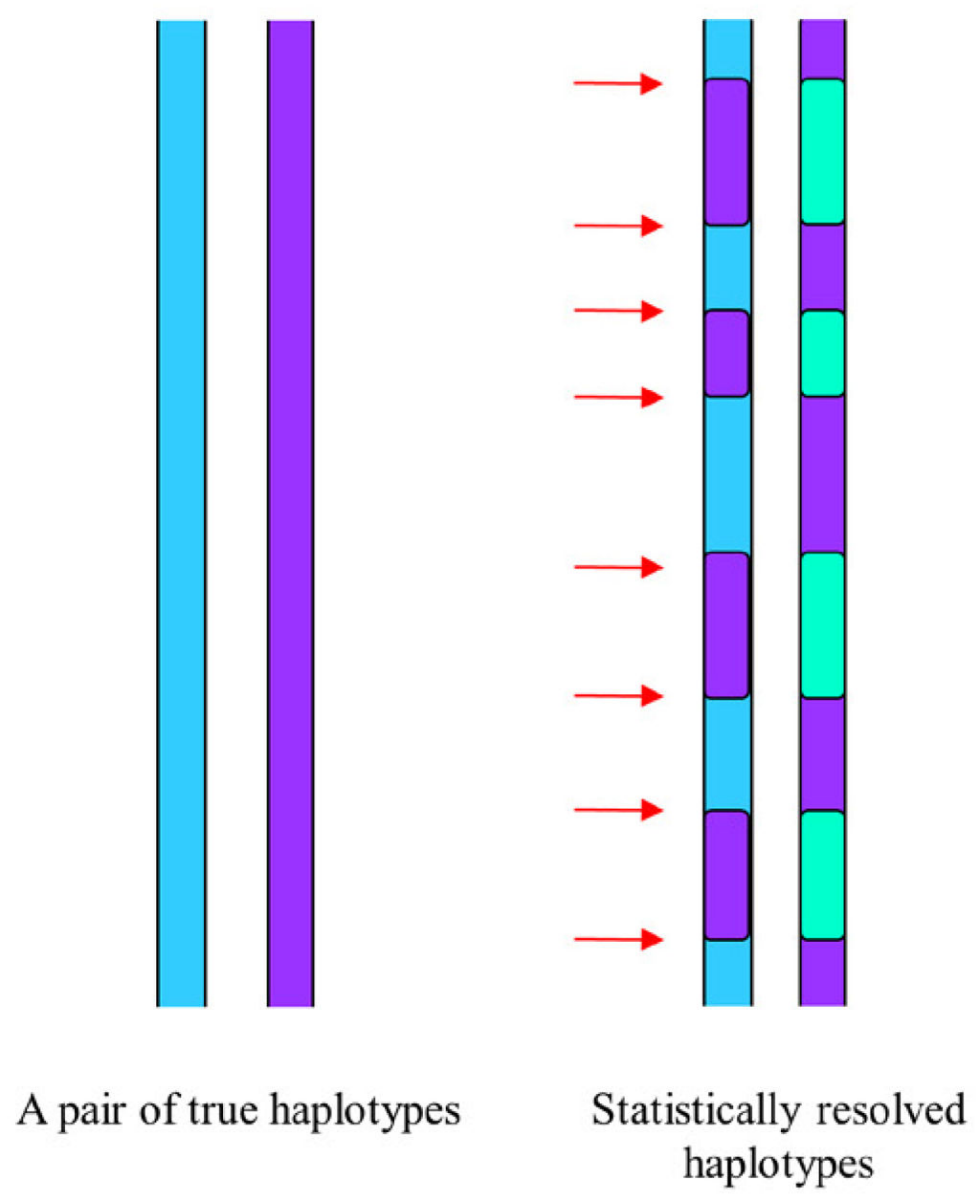
An illustration of switching errors in statistically resolved haplotypes The true chromosomal haplotypes for two homologous chromosomes of an individual are shown on the left. The statistically resolved haplotypes are shown on the left. The red arrows indicate the positions of switching errors. The statistical haplotypes are usually correct within a short-range genomic region between two adjacent switching errors. Although statistically resolved haplotypes contain switch errors, when the sliding window size is significantly smaller than the average size of haplotype segments between two adjacent switching errors, these statistical haplotypes can still be used as reference haplotypes in the data imputation and yield high-quality results.

Until now, statistical inference from genotype data is still the most practical and economical approach for obtaining haplotypes; however, this approach still suffers from ambiguities, low accuracy over a long distance with switching errors (Figure 3) [10,34–36]. Chromosomal segments may be phased correctly but their connections to each other are often incorrect along the entire chromosomes. Such errors can occur many times along the entire length of chromosomes. Moreover, it cannot predict where the switching errors occur along the chromosomes (ambiguities). Even so, it was demonstrated that the reference panel composed of statistically resolved haplotypes can successfully yield the high-quality imputation results that is similar to the results obtained with the reference panel composed of molecular haplotypes [31,32]. We investigated the reason underlying this observation, and found that the size of sliding windows is usually much smaller than the segmental sizes of haplotype stretches between switching errors in the statistical phasing results; due to the relatively high accuracy within each haplotype stretch in the statistically resolved haplotypes, the imputation can extract correct information from each sliding window. This strategy can make a good use of existing big data and overcome the caveats (switching errors and ambiguities) of the big data of unphased genotypes; it becomes a powerful approach in addition to the pooling strategy and the internal reference strategy for haplotype imputation.

## The importance to determine long-range haplotypes in medicine

**Figure 4:**
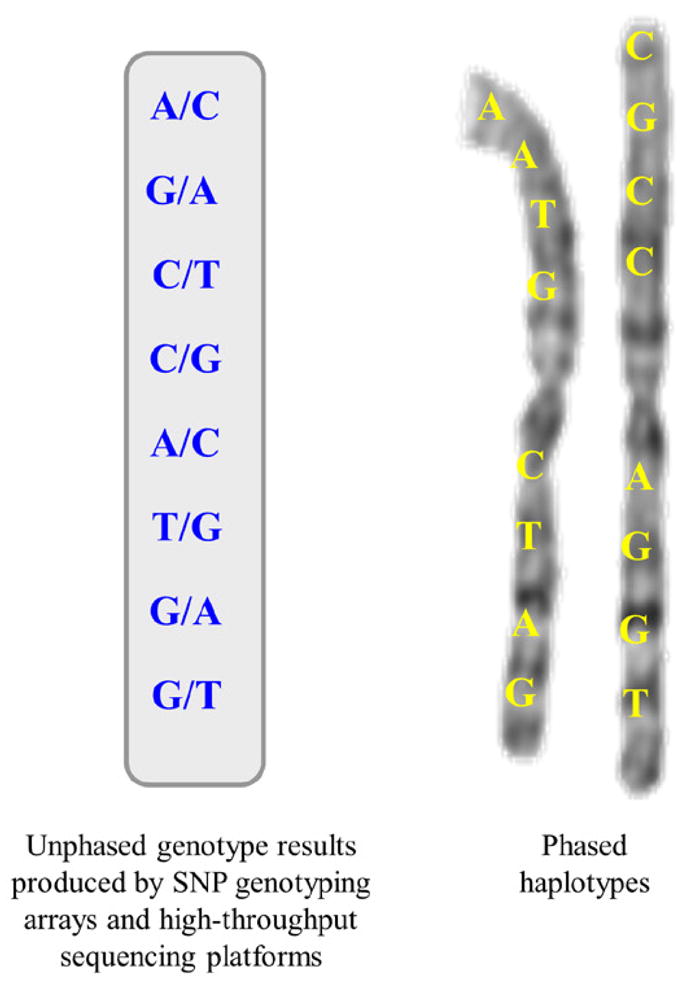
An illustration of genotypes (unphased nucleotide sequences) haplotypes (phased nucleotide sequences).

Haplotype refers to a group of alleles inherited on each of the homologous chromosomes (Figure 4). Haplotype is related to molecular functions [37,38]. Humans are diploid, with two sets of homologous chromosomes in each somatic cell, one inherited from mother, one from father. Although those two copies of homologous chromosomes share a high similarity in human genome, their nucleotide sequences are different and the gene functions on these chromosomes are not similar [39–47]. The high-throughput sequencing technologies can only provide the information of primary sequential orders of nucleotides; they cannot provide the other half of genetic information in human genome, the structural conformations of nucleotides. Without the phase information, all of these ‘personal genomes’ are incomplete, and should essentially be regarded as rough draft genomes [10].

**Figure 5:**
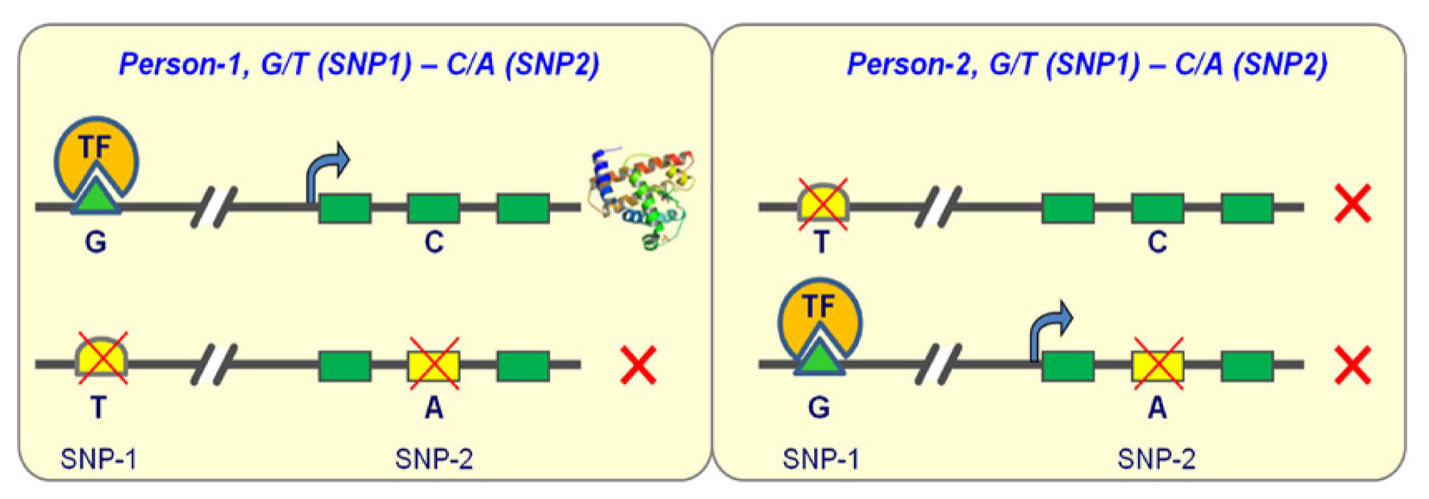
An illustration of the importance of haplotype information for functional interpretations of the genetic variants This illustration shows two individuals with exactly the same genotypes but different haplotypes. In this model, SNP-1 (G/T) is a cis-acting regulatory SNP in an enhancer, in which Allele-G is functional but Allele-T is not. SNP-2 (C/A) is a missense coding SNP in an exon of this gene, in which Allele-C is functional but Allele-A is not. Person-1 can produce this protein because one of his two chromosomal gene copies is normal without the disruptive alleles; Person-2 cannot produce this protein because neither of his gene copies contains two functional alleles. In this hypothetical model, the cis-conformation will determine whether an individual will be sick or not.

As stated by the *nature* special issue released in the February 2015 on epigenome roadmap and the ENCODE strategic planning meeting (ENCODE and Beyond) held in March 2015, increasing evidence showed that not just one or two genetic elements dictate the gene activity and functions; instead, cis-interactions of multiple elements dictate gene activity [48–50]. It has been revealed that extensive allelic imbalance events are associated with cis-regulatory elements [51]. Long-range cis-interactions have been systematically examined with chromosome conformation capture (3C) [52], chromosome conformation capture carbon copy (5C) [53] and Hi-C technique [52]. It has been observed that only 7% of looping interactions between gene expression and promoter-enhancers are with the nearest gene, indicating that genomic proximity is not a simple predictor for long-range interactions [53,54]. It is believed that cis-regulatory mutations affect a broad range of morphological, physiological and neurological phenotypes. Classic examples include the HLA typing (human leukocyte antigen) on the chromosome 6p21, which is associated with more than 100 different diseases, mostly autoimmune diseases such as type I diabetes, rheumatoid arthritis, psoriasis, and atopic asthma. Long range haplotyping is required [55]. The unphased genotype data is sufficient for those rare diseases caused by single mutations; but only haplotypes can unveil the secrets underlying the common diseases involving cis-interactions among multiple genetic variants. Phenotypic effects of genetic variants are best understood in terms of multi-locus haplotypes rather than single-locus variants because the configuration may have a tremendous impact on gene functions as illustrated by Figure 5. In order to study the biological, physiological and pathological functions of genetic variations in human genome, it is necessary to decipher these cis-interactions and their synergy rather than studying them one by one [53,54]. It has been widely accepted that haplotype information will be extremely valuable in a wide spectra of applications from academic research, to clinical diagnosis, prevention, treatment, and pharmaceutical industry, but the wide clinical applications of haplotype-based diagnosis await new advances at present.

## Supplementary Material

table

## Abbreviations

ASW: African ancestry in Southwest USA
CEU: Utah residents with Northern and Western European ancestry from the CEPH collection
CHB: Han Chinese in Beijing, China
GIH: Gujarati Indians in Houston, Texas
JPT: Japanese in Tokyo, Japan
MKK: Maasai in Kinyawa, Kenya
YRI: Yoruba in Ibadan, Nigeria
AADM: The Africa America Diabetes Mellitus Study
ENCODE: The Encyclopedia of DNA Element Consortium
GALA: Gene-Environment Studies of Asthma in Hispanic/Latino Children
HGDP: Human Genome Diversity Project
KGP: 1,000 Genomes Project
DHSs: DNase I hypersensitive sites
GWAS: Genome-Wide Association Studies
HLA: Human Leukocyte Antigen
SNP: Single-Nucleotide Polymorphisms
TSSs: Transcriptional start sites
